# Psychometric evaluation of the endometriosis impact questionnaire (EIQ) in an Iranian population

**DOI:** 10.1186/s12905-024-02975-7

**Published:** 2024-02-20

**Authors:** Mojgan Mirghafourvand, Vahid Ghavami, Maryam Moradi, Khadijeh Mirzaii Najmabadi, Sanaz Mollazadeh

**Affiliations:** 1 Social Determinants of Health Research Center, Tabriz University of Medical Sciences, Tabriz, Iran; 2 Department of Biostatistics, School of Health, Social Determinants of Health Research Center, Mashhad University of Medical sciences, Mashhad, Iran; 3 Department Of General Practice, School Of Public Health and Preventive Medicine, Faculty Of Medicine, Nursing and Health Science, Monash University, Melbourne, Australia; 4 Nursing and Midwifery Care Research Center, Mashhad University of Medical Sciences, Mashhad, Iran; 5 Department of Midwifery, Research Student Committee, Mashhad University of Medical Sciences, Mashhad, Iran

**Keywords:** Translation, Psychometrics, Validity, Reliability, Endometriosis impact questionnaire, Endometriosis

## Abstract

**Background:**

Endometriosis is a benign and chronic gynecological estrogen-dependent disease. Considering the prevalence and the importance of measuring the long-term effects of endometriosis in affected women’s lives t the EIQ scale was designed and psychometrically analyzed in English in Australia, in three recall periods (last 12 months, 1 to 5 years ago and more than 5 years ago). It has never been used in Iran and its validity and reliability have not been assessed either. Therefore, the present study aimed to translate and investigate the psychometric properties of the EIQ.

**Methods:**

In this study, 200 women were selected through random sampling in 2022. After forward and backward translation, the face validity, content validity, and construct validity of EIQ (through Corrected Item-Total Correlation) were examined. To assess the reliability of the scale, both internal consistency (Cronbach’s alpha) and test-retest stability methods were employed.

**Results:**

Impact Score with a score above 1.5 was approved. CVI and CVR values of the EIQ tool were 0.97 and 0.94, respectively. The Item to total Correlation confirmed the construct validity of all seven dimensions of the tool, more than the cut-off (0.3) except lifestyle. Cronbach’s alpha coefficient and Intra Correlation Coefficient (ICC) were acceptable for all dimensions.

**Conclusion:**

The Persian version of EIQ is a valid and reliable scale. This tool is valid and reliable for investigating the long-term impact of endometriosis in Iranian society.

## Introduction

Endometriosis is a prolonged, benign, and progressive inflammatory gynecological estrogen-dependent disease. It is defined as the existence of endometrial glands and stroma in a place other than the endometrial cavity of the uterus, causing a chronic inflammatory reaction in the pelvis [1]. The most common replacement sites in the pelvic cavity include the ovary, uterosacral ligament and dead end of Douglas, cervix, sigmoid colon, and pelvic peritoneum [2–4]. It is estimated that endometriosis affects one out of ten women during their reproductive years (15 to 49 years old) [5]. Endometriosis affects between 2 and 10% (190 million) of women and girls of reproductive age worldwide, but its prevalence in infertile women can even be up to 33% [6]. Women suffering from this disease suffer from related symptoms such as infertility, periodic and non-periodic abdominal pain, painful menstruation, bloating, diarrhea or constipation, painful intercourse, and painful urination, painful defecation [7, 8]. The etiology of endometriosis is complex and multifactorial [6]. The exact cause of endometriosis is still unclear, but backward menstruation is widely accepted as an effective factor in this disease [9, 10]. Unfortunately, even though life with endometriosis is very difficult for many patients, the problems of these patients have not been given much attention, and affected women suffer from the harmful effects of this disease for a long time [2].

The EIQ questionnaire was designed and psychometrically analyzed in English by Moradi et al. (2019) in Australia, and it has been shown that the EIQ is a valid and reliable tool for measuring the impact of endometriosis on women’s lives with a long-term perspective [11]. The purpose of this tool is to measure the long-term effects of chronic endometriosis on various aspects of the life of women with endometriosis. This questionnaire contains 63 items that measure the impact of the disease on various aspects of the affected women’s lives in 8 dimensions, including the impact of the disease on physical, social, psychological, marital intimacy and sexual relations, fertility, occupational, and economic, education, lifestyle aspects and it examines the impacts of endometriosis in three time periods (last 12 months, 1 to 5 years ago and more than 5 years ago) [11]. To design this tool, a qualitative study with a thematic analysis approach was conducted in phase 1, and then a cross-sectional study was conducted in Phase 2.

Other standard instruments such as EHP-30^1^ (2001) or EHRQ^2^ (2021) examine the quality of life in these patients during the last four weeks. This questionnaire with 30 items includes five scales of pain, control and disability, emotional well-being, social support, and self-image. Six central section’s consisting of 23 questions measure sexual intercourse, work, relationship with children, feelings about the medical profession, treatment, and infertility [12].

Also, Endometriosis Reproductive Health Questionnaire (ERHQ) was conducted in Iran in 2021, has 35 items and 4 dimensions, physical problems (9 items, questions 1–9), mental-psychological problems (12 items, questions 10–21), instability in married life (8 items, questions 22–29) coping strategies (6 items, questions 30–35) [13]. But due to the chronic and recurring nature of this disease, some of the effects of the disease can be ignored with a short-term perspective. EIQ is the first questionnaire that measures multidimensional effects with a long-term perspective. Validation of a tool includes collecting empirical evidence about its use. Compared to EHP-30, EIQ has two new subscales of education and lifestyle, while EHP-30 has subscales of communication with children, medical professionals, and therapy [11].

Studies have recommended the need to provide appropriate measures and programs to promote health in patients with endometriosis, and the first step is to measure the impacts of the disease on the lives of affected women. Therefore, considering the prevalence and importance and chronic nature of endometriosis, the importance of measuring the impacts of the disease in affected women in order to measure and design appropriate interventions; also the lack of valid and reliable tools to measure prolonged impacts of endometriosis in Iranian women, the present study aimed to “translate and psychometrically analyze the Persian version of the EIQ Questionnaire”.

## Methods

### Study participants

The present study was confirmed by the Ethics Council of Mashhad University of Medical Sciences (ethics code: IR.MUMS.NURSE.REC. 4,010,521). This descriptive-analytical cross-sectional study, which was done in 2022 from August to November, recruited women of reproductive age (15–49 years), with endometriosis who were referred to the endometriosis clinic of Imam Reza Hospital in Mashhad-Iran.

Inclusion criteria included women of reproductive age (15–49 years old) with endometriosis, endometriosis diagnosis by open surgery or laparoscopy or histological diagnosis or the presence of endometrioma cyst and diagnosis by ultrasound and MRI and confirmed by a gynecologist, at least one year after diagnosed with endometriosis, Iranian women, married women, literate and able to answer questions, not menopausal (stopping menstruation for more than one year), not suffering from other major diseases including mental disorders including depression, eating disorders and obesity, polycystic ovary syndrome (PCOS), infertility, insomnia, schizophrenia and chronic diseases including diabetes, kidney disease and rheumatology, absence of cancer and any life-threatening diseases according to the reports of research units. Exclusion criteria included incomplete completion of the questionnaire by not answering more than 10% of the questions.

### Sample size

For construct validity and factor analysis, Hair et al. state that the sample size should be more than 100 samples, and according to the strategy proposed by Hair et al. the minimum required sample size is 3 samples and the maximum is 20 samples per item [14]. Considering the 63 questions of the EIQ questionnaire, the total sample size was determined approximately 200 women. The sample size was different in each stage of psychoanalysis. For content validity: 10 experts; for face validity: 10 qualified women with endometriosis; to evaluate internal consistency: 20 women with endometriosis. For reliability (retest): 20 women with endometriosis; for construct validity (confirmatory factor analysis): 200 women with endometriosis.

### Introducing the tool

The Endometriosis Impact Questionnaire (EIQ) is a self-report questionnaire that examines how endometriosis has affected women’s lives over the three recall periods including ‘last 12 months’, ‘1 to 5 years ago’ ,and ‘more than 5 years ago’. EIQ items are rated on a 5-point Likert scale, ranging from 0 (not at all) to 4 (very much), with the additional option of 9 (not applicable). Each item contributes equally and higher scores indicate a greater impact. The EIQ was developed in Australia and a psychometric evaluation was conducted, using face, content, construct (factor analysis), concurrent validity, and reliability (internal consistency and test-retest reliability). the study by Moradi et al. used a methodological design that involved the development and evaluation of data collection instruments, scales, or techniques. To evaluate construct and concurrent validity and reliability, a cross-sectional study was conducted via a web-based survey. All data were analyzed using SPSS version 20, and probability values of *p* < 0.05 were considered to be statistically significant [11].

### Translation process

Because the translation and psychometry of the tool in question have not been done in Iran, in this study, the translation and psychometry of the Persian version of this tool were done. In the first step, translation (Forward & Backward Translation), the desired tool was translated from English to Farsi by a fluent colleague in both languages (at least two people). In the second step, the primary translations were combined into a single translation. In the third step, the final translated version was returned from the target language to the original language. In the fourth step, the translated version was revised from the target language to the original language. In this step, literal translation was not meant, but semantic translation will be done. The meanings hidden and present in the original version and its transfer to the Persian language were considered instead of the exact translation of the words. The questions and words of the original questionnaire must have the same meaning as the translated version [15].

### Data collection

The researcher was presented at the endometriosis clinic of Mashhad University of Medical Sciences, located at Imam Reza Hospital. Eligible women were invited to participate in the study by referring to the medical records of women with endometriosis available in the endometriosis clinic. After introducing herself to the women and explaining the purpose of the study, the researcher invited eligible and willing women to participate in the study after obtaining written and informed consent. Women entered the study after an explanation about the study and obtaining informed consent.

### Data analysis

After collecting the data, it was coded and the data was entered into the SPSS version 21 software. Descriptive statistics including frequency (percentage) and mean ± standard deviation were used to describe socio-demographic characteristics. To check construct validity, the Corrected Item-Total Correlation was used. To verify the reliability of the current scale, Cronbach’s alpha methods were utilized to calculate internal consistency and test-retest reliability was determined through ICC analysis.

### Face validity

For face validity, quantitative and qualitative approaches were used. The quantitative approach was evaluated by calculating the impact score, and the qualitative approach was based on the opinions of the expert committee and target groups’ views. Questionnaire items in the face validity form include the first part (qualitative evaluation), checking in terms of difficulty levels, irrelevance, and ambiguity. The second part (quantitative evaluation) was included in calculating the impact score, checking the importance of the items based on a 5-point Likert scale (completely important, important, moderately important, slightly important, and not important). Then, the convenience sampling questionnaire was given to 10 eligible women and their husbands. Lastly, the score of each item was calculated using the following formula: Impact Score = Frequency (%) × Importance. Finally, the items with an impact score of more than 1.5 were accepted.

(Frequency: percentage of women who responded to a specific Likert in the desired item. Importance: Likert number chosen by women [16].

### Content validity

Quantitative and qualitative methods were used to evaluate content validity. In the qualitative component, ten specialists across midwifery and reproductive health were asked to examine and provide corrective opinions on the translation of each question concerning its grammar, appropriateness of wording, and th sentence structure. After collecting the experts’ evaluations, the required changes were given in the tool. Content validity was quantitatively calculated based on the opinions of experts and by calculating two indexes: content validity ratio (CVR = Content Validity Ratio) and content validity index (CVI = Content Validity Index). The content validity index of the questions was assessed regarding relevance, clarity, and simplicity based on a 4-point Likert scale.

### Construct validity

Due to the large number of missing values of the scoring of the questionnaire; Confirmatory factor analysis was not feasible for evaluating construct validity. Therefore, the item-to-total correlation method was employed for this purpose. The cut-off point for this method was set at 0.3, which implies that any variable with a corrected item-total correlation value less than 0.3 should be eliminated [17–19].

### Tool Reliability

For the reliability of the instrument, internal consistency was used by calculating Cronbach’s alpha with the SPSS 21 software. Also, to determine the repeatability, the test-retest method through determining the intra-class correlation coefficient (ICC) was used. Twenty women with endometriosis answered the questions of the Persian version two times with an interval of 2 weeks.**Results**.

### Participants’ characteristic

In this study, 200 women with endometriosis were assessed. The results of Table 1 show the socio-demographic characteristics of participants. The participants’ mean age was 35.6 years (SD: 6.2). In terms of education level, the number (percent) of academic degrees was 123 (61.5%). More than half of women (58.5%) were housewives. In terms of monthly income adequacy, two thirds of women (69%) had a sufficient income for living expenses.

**Table 1:** Characteristics of the study participants for Construct validity (*n* = 200)

Characteristics	Number (Percentage)
**Age (Year)**	35.6 (6.2) *
**Level of Education**	
Elementary/Secondary school	20 (10.0)
High School/Diploma	57 (28.5)
Academic	123 (61.5)
**Job**	
Housewife	117 (58.5)
Employed	83 (41.5)
**Adequacy of monthly income**	
Less than living expenses	43 (21.5)
Equal of living expenses	138 (69.0)
More than living expenses	19 (9.5)

*Mean (SD)

### Face and content validity

In the face validity review, all items were described as appropriate and without ambiguity and difficulty and received a minimum score of 1.5, except items 61, 62, and 63 which scored less than 1.5. Also, in the content validity evaluation, all items obtained the minimum acceptable value of CVR and CVI. CVR for the whole tool was 0.94 and CVI for the whole tool was 0.97 (Table 2).

**Table 2:** Impact coefficient, index and content validity ratio of the items of the Endometriosis Impact Questionnaire (EIQ)

Items	Impact factor* n = 10 women	CVI^†^ CVR^‡^ *n* = 10 experts	
EIQ 1	4.5	1	1
EIQ 2	3.6	1	1
EIQ 3	3.2	1	1
EIQ 4	3.6	1	1
EIQ 5	2.7	0.96	1
EIQ 6	3.4	1	1
EIQ 7	2.3	0.83	0.80
EIQ 8	4	1	1
EIQ 9	2.8	1	1
EIQ 10	1.7	1	1
EIQ 11	3.2	1	1
EIQ 12	3.5	1	1
EIQ 13	2.5	0.96	1
EIQ 14	3	1	1
EIQ 15	3.3	0.86	0.80
EIQ 16	6	0.90	0.80
EIQ 17	4.2	1	1
EIQ 18	2.8	1	1
EIQ 19	2.4	1	1
EIQ 20	1.7	0.90	0.80
EIQ 21	3.4	0.90	0.80
EIQ 22	2.4	0.90	0.80
EIQ 23	2.7	0.90	0.80
EIQ 24	3.6	1	1
EIQ 25	4.3	1	1
EIQ 26	3.2	1	1
EIQ 27	2.7	0.96	1
EIQ 28	2.2	1	1
EIQ 29	3.8	1	1
EIQ 30	2.6	1	1
EIQ 31	2.1	1	1
EIQ 32	2.2	1	1
EIQ 33	1.9	1	1
EIQ 34	2	1	1
EIQ 35	3	1	1
EIQ 36	3.2	1	1
EIQ 37	2.7	1	1
EIQ 38	3.3	1	1
EIQ 39	2.7	1	1
EIQ 40	3.6	0.86	0.80
EIQ 41	4	1	1
EIQ 42	4.1	0.90	0.80
EIQ 43	3.4	1	1
EIQ 44	1.9	0.90	0.80
EIQ 45	2.7	1	1
EIQ 46	2.1	1	1
EIQ 47	2.2	1	1
EIQ 48	2.7	1	1
EIQ 49	1.9	0.90	0.80
EIQ 50	1.9	1	1
EIQ 51	2	1	1
EIQ 52	1.9	1	1
EIQ 53	2.7	0.90	0.80
EIQ 54	3.1	1	1
EIQ 55	2.7	1	1
EIQ 56	2.8	1	1
EIQ 57	1.9	0.93	1
EIQ 58	1.9	1	1
EIQ 59	1.9	0.96	1
EIQ 60	1.7	0.96	0.80
EIQ 61	0.18	0.83	0.80
EIQ 62	1.1	0.86	0.80
EIQ 63	0.44	0.90	0.80
Overall score of EIQ		0.97	0.94

^*^Impact Score, ^†^Content Validity Index, ^‡^Content Validity Ratio

The whole tool and its dimensions had a minimum standard of internal consistency above 0.7. ICC for the physical, psychological, social, sexual, occupational, and financial effects and education dimensions was found to be above 0.7, which indicates an acceptable agreement for the questionnaire (Table 3).

**Table 3:** Internal consistency and retest stability of endometriosis effects questionnaire with 20 women with endometriosis

Dimensions of endometriosis effects	Last 12 months		Last 1–5 years		Last 5 years	
	Cronbach’s alpha	ICC (95% CI) *	Cronbach’s alpha	ICC (95% CI) *	Cronbach’s alpha	ICC (95% CI) *
Physical effects	0.90	0.89 (0.86–0.91)	0.88	0.86 (0.89 − 0.84)	0.73	0.94 (0.93–0.95)
Psychological effects	0.81	0.93 (0.94 − 0.91)	0.87	0.95 (0.94–0.96)	0.87	0.98 (0.98 − 0.97)
Social effects	0.91	0.90 (0.92 − 0.88)	0.93	0.94 (0.92–0.95)	0.93	0.97 (0.97 − 0.96)
Sexual relationships	0.92	0.81 (0.75–0.86)	0.84	0.94 (0.92–0.95)	0.93	0.96 (0.95–0.97)
Reproductive	0.99	0.78 (0.71–0.84)	0.95	0.77 (0.69–0.84)	0.93	0.86 (0.82–0.90)
Occupational and financial	0.72	0.93 (0.91–0.95)	0.74	0.94 (0.93–0.96)	0.70	0.96 (0.95–0.97)
Education	0.85	0.88 (0.78–0.95)	0.82	0.94 (0.97 − 0.89)	0.84	0.96 (0.98 − 0.92)
Lifestyle	0.93	(0.92–0.95)0.94	0.94	(0.96 − 0.94) 0.96	0.95	(0.94–0.96) 0.94

*Intra-class Correlation Coefficient (95% Confidence Interval)

### Construct validity

In the construct validity review of EIQ, all items demonstrated a minimum corrected item-total correlation value of 0.3, except for items 61, 62, and 63 (as shown in Table 4).

**Table 4:** Construct validity of the items of the Endometriosis Impact Questionnaire (EIQ) with 200 women with endometriosis

Items	Last 12 months		Last 1–5 years		Last 5 years	
	Cronbach’s alpha	Item-Total Correlation	Cronbach’s alpha	Item-Total Correlation	Cronbach’s alpha	Item-Total Correlation
EIQ 1	0.88	0.68	0.87	0.64	0.85	0.52
EIQ 2	0.88	0.56	0.87	0.54	0.85	0.62
EIQ 3	0.88	0.55	0.87	0.51	0.85	0.35
EIQ 4	0.88	0.30	0.87	0.35	0.85	0.37
EIQ 5	0.88	0.67	0.87	0.61	0.85	0.67
EIQ 6	0.88	0.67	0.87	0.65	0.85	0.58
EIQ 7	0.88	0.58	0.87	0.57	0.85	0.53
EIQ 8	0.88	0.67	0.87	0.62	0.85	0.66
EIQ 9	0.88	0.73	0.87	0.69	0.85	0.73
EIQ 10	0.88	0.72	0.87	0.65	0.85	0.65
EIQ 11	0.88	0.48	0.87	0.54	0.85	0.55
EIQ 12	0.88	0.49	0.87	0.45	0.85	0.38
EIQ 13	0.88	0.38	0.87	0.48	0.85	0.37
EIQ 14	0.94	0.72	0.94	0.73	0.96	0.79
EIQ 15	0.94	0.52	0.94	0.55	0.96	0.74
EIQ 16	0.94	0.62	0.94	0.70	0.96	0.78
EIQ 17	0.94	0.68	0.94	0.73	0.96	0.81
EIQ 18	0.94	0.74	0.94	0.81	0.96	0.79
EIQ 19	0.94	0.78	0.94	0.76	0.96	0.76
EIQ 20	0.94	0.57	0.94	0.51	0.96	0.55
EIQ 21	0.94	0.78	0.94	0.78	0.96	0.81
EIQ 22	0.94	0.65	0.94	0.59	0.96	0.74
EIQ 23	0.94	0.56	0.94	0.60	0.96	0.65
EIQ 24	0.94	0.73	0.94	0.75	0.96	0.84
EIQ 25	0.94	0.72	0.94	0.78	0.96	0.88
EIQ 26	0.94	0.67	0.94	0.68	0.96	0.77
EIQ 27	0.94	0.77	0.94	0.80	0.96	0.83
EIQ 28	0.94	0.57	0.94	0.62	0.96	0.64
EIQ 29	0.94	0.74	0.94	0.81	0.96	0.83
EIQ 30	0.90	0.82	0.90	0.82	0.89	0.82
EIQ 31	0.90	0.80	0.90	0.78	0.89	0.77
EIQ 32	0.90	0.77	0.90	0.78	0.89	0.80
EIQ 33	0.90	0.76	0.90	0.76	0.89	0.67
EIQ 34	0.81	0.54	0.83	0.60	0.88	0.72
EIQ 35	0.81	0.40	0.83	0.46	0.88	0.51
EIQ 36	0.81	0.67	0.83	0.68	0.88	0.77
EIQ 37	0.81	0.69	0.83	0.69	0.88	0.74
EIQ 38	0.81	0.65	0.83	0.65	0.88	0.73
EIQ 39	0.81	0.42	0.83	0.44	0.88	0.52
EIQ 40	0.81	0.48	0.83	0.61	0.88	0.71
EIQ 41	0.82	0.63	0.86	0.72	0.92	0.84
EIQ 42	0.82	0.72	0.86	0.77	0.92	0.91
EIQ 43	0.82	0.68	0.86	0.75	0.92	0.79
EIQ 44	0.93	0.84	0.91	0.76	0.89	0.57
EIQ 45	0.93	0.84	0.91	0.81	0.89	0.69
EIQ 46	0.93	0.67	0.91	0.65	0.89	0.65
EIQ 47	0.93	0.64	0.91	0.56	0.89	0.57
EIQ 48	0.93	0.71	0.91	0.65	0.89	0.70
EIQ 49	0.93	0.84	0.91	0.81	0.89	0.58
EIQ 50	0.93	0.77	0.91	0.65	0.89	0.70
EIQ 51	0.93	0.74	0.91	0.68	0.89	0.53
EIQ 52	0.93	0.69	0.91	0.69	0.89	0.54
EIQ 53	0.93	0.81	0.91	0.76	0.89	0.74
EIQ 54	0.93	0.46	0.91	0.43	0.89	0.65
EIQ 55	0.96	0.59	0.90	0.35	0.92	0.59
EIQ 56	0.96	0.98	0.90	0.96	0.92	0.94
EIQ 57	0.96	0.96	0.90	0.77	0.92	0.75
EIQ 58	0.96	0.91	0.90	0.79	0.92	0.78
EIQ 59	0.96	0.96	0.90	0.87	0.92	0.95
EIQ 60	0.96	0.87	0.90	0.74	0.92	0.71
EIQ 61	0.12	0.10	-0.4	-0.1	-0.1	-0.1
EIQ 62	0.12	0.10	-0.4	-0.2	-0.1	-0.0
EIQ 63	0.12	-0.02	-0.4	-0.1	-0.1	-0.1

## Discussion

The present research was conducted to determine the psychometric properties of the EIQ for Iranian women affected by endometriosis. It was demonstrated that the Persian version of this scale is valid and reliable tool to measure the impact of endometriosis on women’s lives with a long-term perspective. Validity was assessed and confirmed using face validity (qualitative and quantitative), content validity (qualitative and quantitative), and construct validity (item to total correlation). The reliability of the tool was also examined and approved through internal consistency (Cronbach’s alpha coefficient) and test-retest stability.

The EIQ with eight dimensions including physical, psychological, social, marital intimacy and sexual relations, reproductive, occupational and financial, and education aspects had a proper face and content validity except for the lifestyle aspects. Face validity means that items comprehensively covers the different components of endometriosis impacts to be measured and content validity indicates that items are sensible, appropriate, and relevant to the women who use the measure [20].

The results of the psychometric evaluation of the Endometriosis Impact Questionnaire (EIQ) in an Iranian population, showed that the lifestyle dimension with 3 questions that raised the use of alcohol and drugs to adapt to the disease impact, scored below the minimum cut-off in the construct validity and impact score. The reason for this result could be related to cultural differences and beliefs of Iranian women. In Iranian women’s culture, the use of these substances is considered taboo and it is considered disrespectful for a woman.

According to the results of previous studies, caffeine, alcohol, and smoking can cause changes in the synthesis of known sex steroids (SHBG), which may affect the risk of hormone-related diseases, such as endometriosis [21–25]. In contrast the results of Hemmert et al.’s study which is unique in its capture of lifestyle exposures before incident endometriosis diagnosis, largely found no association between alcohol, caffeine, smoking, and physical activity and risk of endometriosis [24]. Also, studies demonstrated the relationship between certain foods or lifestyle modifications is limited. The result of the Manaker et al. study showed that increasing consumption of certain fruits, omega-3 fatty acids, and dairy foods may reduce the risk of developing endometriosis. Dietary and lifestyle modifications and how they are related to endometriosis risk factors and/or symptoms associated with endometriosis are discussed [25].

The EIQ (2014) is the first questionnaire to measure the multi-dimensional impacts of endometriosis with a long-term perspective. Considering its recurring nature, symptoms may continue despite seemingly adequate treatment [26]. Also, the other standard tools such as (EHP-30 or ERHQ) are available. For example the ERHQ (2021) was designed in Iran. ERHQ is a new, valid and reliable patient-generated instrument to measure the reproductive health of women with endometriosis. The number of items in the EHRQ questionnaire is 35. Four factors explained the questionnaire’s factor structure: physical problems (9 items), psychological problems (12 items), counteracting strategies (6 items), and instability of marital life (8 items) [13].

 [11]. Also, the EHP-30 is the disease-specific 30-items questionnaire to measure the HRQoL^3^ with the strongest validity evidence. The EHP-30 has subscales of relationship with medical professionals, treatment, and children [27].The EIQ has two new subscales of education and lifestyle, comparedwith the EHP-30 [12]. Both EHP-30 and ERHQ or other tools measure the effect of endometriosis during last four weeks. So some impacts could be missed by only looking at the last four weeks, because of chronic nature of endometriosis. Also, other questionnaires are not able to investigate occupational goals, loss of job or promotion opportunities, address women’s regrets from living with endometriosis, or measure the impacts on a woman who lost her sexual-intimate relationship. Future studies should be conducted to measure the impact of endometriosis on lifestyle, as well as education and work [28, 29].

This questionnaire with 30 items includes five scales of pain, control and disability, emotional well-being, social support, and self-image. Six central section’s consisting of 23 questions measure sexual intercourse, work, and relationship with children, feelings about the medical profession, treatment, and infertility (124). However, due to the chronic and recurrent nature of this disease, some of the effects of the disease can be ignored in the past four weeks. EIQ is the first questionnaire that measures multidimensional effects with a long-term perspective. Validation of a tool includes collecting empirical evidence about its use. Similar to EIQ, instruments such as ETSQ and EPBD were designed from focus group discussions and interviews with patients. EHP-30 items were designed based on open exploratory interviews with 25 women with endometriosis. The process used to validate the EIQ was somewhat similar to that used for the ECQ, however, the development processes were different. Compared to the EHP-30, the EIQ has two new subscales of education and lifestyle, while the EHP-30 has subscales of communication with children, medical professionals, and therapy (123).

### Strengths and limitations

This is the first approved translation and adaptation of the EIQ in Iran as a Middle-Income Country and it performs in the same manner as the Australian tool, except for lifestyle dimension. It could evaluate the multi-dimensional impacts of endometriosis to provide detailed information in population health surveys and to compare different management options or different areas and stages of patients’ lives. It could consider all three recall periods or each period independently because each has satisfactory validity and reliability. The total score for each dimension at three recall periods, for all dimensions at each recall period, and the total impact score could be calculated. Combining the scores will depend on the research objectives. The recall period of ‘last 12 months’, could be used to investigate outcomes in clinical trials. It could help patients communicate with health professionals and be useful to guide the development of an individualized disease management plan. It could also be used to assess of patients’ needs or as a burden estimation to provide information for making health policy decisions to improve services for affected women’s lives. The limitations of this study include that the questionnaire contained questions that led to a large number of empty responses because the participants did not have the conditions at that time to answer those questions. This limitation resulted in a high amount of missing data, preventing the conduct of confirmatory factor analysis and divergent validity and convergent validity in construct validity.

## Conclusion

The results showed that the EIQ is a valid and reliable tool to measure the impacts of endometriosis on different aspects of Iranian women’s lives with a long-term view. It can be used by researchers and health providers to provide a better understanding of the impact of endometriosis on various aspects of reproductive health over time and to meet the needs of patients with the disease. In future studies, it is recommended to conduct studies with a more robust methodology and larger sample sizes in various communities and other countries as well as different languages with different sociocultural contexts to achieve more generalizable results and to make multinational studies possible.

## Data Availability

The datasets generated and/or analyses during the current study are available from the corresponding author on reasonable request.

## Footnotes

1 Endometriosis health profile.

2 Endometriosis reproductive health questionnaire.

3 Health related quality of life.
