# Correction to: Psychometric evaluation of the endometriosis impact questionnaire (EIQ) in an Iranian population

**DOI:** 10.1186/s12905-024-03054-7

**Published:** 2024-04-08

**Authors:** Mojgan Mirghafourvand, Vahid Ghavami, Maryam Moradi, Khadijeh Mirzaii Najmabadi, Sanaz Mollazadeh

**Affiliations:** 1 Social Determinants of Health Research Center, Tabriz University of Medical Sciences, Tabriz, Iran; 2 Department of Biostatistics, School of Health, Social Determinants of Health Research Center, Mashhad University of Medical sciences, Mashhad, Iran; 3 Department Of General Practice, School Of Public Health and Preventive Medicine, Faculty Of Medicine, Nursing and Health Science, Monash University, Melbourne, Australia; 4 Nursing and Midwifery Care Research Center, Mashhad University of Medical Sciences, Mashhad, Iran; 5 Department of Midwifery, Research Student Committee, Mashhad University of Medical Sciences, Mashhad, Iran

**Correction to: BMC Womens Health (2024) 24:1**

10.1186/s12905-024-02975-7.

Following publication of the original article [1], the wrong Supplementary file was originally published with this article; it has now been removed from the original article.

The original article has been corrected.
